# Prolonged administration of the granisetron transdermal delivery system reduces capecitabine plus oxaliplatin regimen induced nausea and vomiting

**DOI:** 10.1186/s12885-024-12616-9

**Published:** 2024-07-18

**Authors:** Cong Wang, Zhansheng Jiang, Jie Zhang, Yan Zhuang, Lining Sun, Jing Zhang, Manman Quan, Lan Lan, Yanwei Li, Bin Wang, Zhanyu Pan, Zhuchen Yan

**Affiliations:** 1 Department of Integrated Traditional & Western Medicine, Key Laboratory of Cancer Prevention and Therapy, Tianjin Medical University Cancer Institute and Hospital, National Clinical Research Center for Cancer, Tianjin’s Clinical Research Center for Cancer, Huan-Hu-Xi Road, Ti-Yuan-Bei, He Xi District, Tianjin, 300060 China; 2 Department of Colorectal Oncology, Key Laboratory of Cancer Prevention and Therapy, Tianjin Medical University Cancer Institute and Hospital, National Clinical Research Center for Cancer, Tianjin’s Clinical Research Center for Cancer, Tianjin, China; 3 Department of Radiation Oncology, Department of Oncology, Shanghai Medical College, Fudan University Shanghai Cancer Center, Fudan University, Shanghai, China; 4 Department of Integrative Oncology, Tianjin Cancer Hospital Airport Hospital, Tianjin, China; 5 Interventional Therapy Department, Key Laboratory of Cancer Prevention and Therapy, Tianjin Medical University Cancer Institute and Hospital, National Clinical Research Center for Cancer, Tianjin’s Clinical Research Center for Cancer, Tianjin, China

**Keywords:** Granisetron, Chemotherapy-induced nausea and vomiting, CapeOX regimen, Transdermal delivery system

## Abstract

**Objective:**

To evaluate the safety and efficacy of the granisetron transdermal delivery system (GTDS) combined with Dexamethasone for preventing chemotherapy-induced nausea and vomiting (CINV) in patients receiving Capecitabine plus Oxaliplatin (CapeOX) therapy.

**Design:**

Open-label, prospective, multi-center phase II trial.

**Setting:**

Three institutions.

**Participants:**

Fifty-four patients scheduled to receive CapeOX chemotherapy.

**Interventions:**

Participants received GTDS (3.1 mg applied to the upper arm 48 h before chemotherapy, replaced on day 5, and discarded on day 12) and Dexamethasone.

**Main outcome measures:**

The primary endpoint was the complete control rate of CINV. Secondary endpoints included the duration of delayed complete control, complete control rate in the acute phase, safety, and quality of life.

**Results:**

The complete control rate for delayed CINV over the entire period (25–480 h) was 72.7% (95% CI 0.57–0.88). The duration of delayed complete control was 17.2 ± 4.5 days, with 51.5% of patients experiencing no nausea during the delayed phase. The complete control rate in the acute phase was 81.8% (95% CI 0.69–0.95). No serious adverse events related to the antiemetic regimen were reported.

**Conclusion:**

Prolonged administration of GTDS is safe and effective for preventing CINV in patients with gastrointestinal malignancies treated with CapeOX.

**Trial Registration:**

ClinicalTrials.gov registry (NCT05325190); registered on October 10, 2021.

**Supplementary Information:**

The online version contains supplementary material available at 10.1186/s12885-024-12616-9.

## Background

Chemotherapy-induced nausea and vomiting (CINV) is a common side effect of chemotherapy. It has a detrimental effect on the quality of life of patients receiving chemotherapy and may cause dose reductions, even to discontinuation of chemotherapy [1]. Chemotherapy causes vomiting via release of multiple neurotransmitters such as 5-hydroxytryptamine (5-HT), substance P, dopamine and histamine [2]. Neurotransmitters after binding to specific receptors are known to stimulate the center of vomiting in the encephalic trunk and, eventually, cause vomiting reflexes. In recent years, by the development of novel antiemetic agents such as 5-HT_3_ receptor antagonists and neurokinin-1 (NK-1) receptor antagonists, a high control rate of CINV was achieved. However, multiple-day chemotherapy induced nausea and vomiting has yet to be overcome. The complex overlapping of acute and delayed emesis is a possible reason.

Capecitabine plus oxaliplatin (CapeOX) is the most common multiple-day chemotherapy regimen in neoadjuvant, adjuvant, and concurrent chemotherapy for gastrointestinal malignancies [3]. However, the complete control rate of delayed nausea and vomiting in patients receiving CapeOX was 48.7% with the combination of a 5-HT_3_ receptor antagonist and a steroid [4]. Furthermore, the completion rate of CapeOX is 83.6%. Full-dose chemotherapy is only 77.8% in clinical practice, and CINV is a major cause [5]. Therefore, it requires a persistent and convenient antiemetic regiment that is both efficacy and practical enough to be easily implemented by patients.

To solve this clinical dilemma, we innovated the antiemetic regiment for CapeOX as prolonged administration of the granisetron transdermal delivery system (GTDS) for 14 days. The GTDS is the first transdermal system approved by the US FDA for CINV [6]. It sustains the release of granisetron at an effective concentration lasting over 7 days, and has a lower incidence of constipation and the corrected QT interval (QTc) prolongation than other drugs delivery methods [7, 8].

This open-label, prospective, multi-center phase II trial aimed to evaluate the use of a combined antiemetic regimen comprising the GTDS (14 consecutive days) and Dexamethasone (DEX) for preventing CINV throughout the chemotherapy course.

## Methods

### Study design

This study was an open-label, prospective single-arm, multi-institutional, phase II study at three institutions in China. The study was conducted in accordance with the Declaration of Helsinki and the Ethical Guidelines for Clinical Studies.

### Patients

Eligible patients were > 18 years, and scheduled to receive CapeOX chemotherapy for the treatment of a confirmed gastrointestinal malignancies. Patients needed to possess an Eastern Cooperative Oncology Group (ECOG) Performance Status of 0–2, and completed questionnaire.

Patients were not eligible if they were scheduled to receive: (i) abnormal clinical hematological and biochemical data; (ii) symptomatic central nervous system malignancies; (iii) requirement of antiemetics at enrollment; (iv) recent (within 21 days) treatment with benzodiazepines, opioids, or glucocorticoid; (v) unstable angina, ischemic heart disease, cerebral hemorrhage or apoplexy; (vi) severe emotional or mental disorders; gastrointestinal obstruction; (vii) women who were breastfeeding, who did not wish to use contraception; (viii) and other patients the investigator judged inappropriate for the study.

### Treatment

**Fig. 1:**
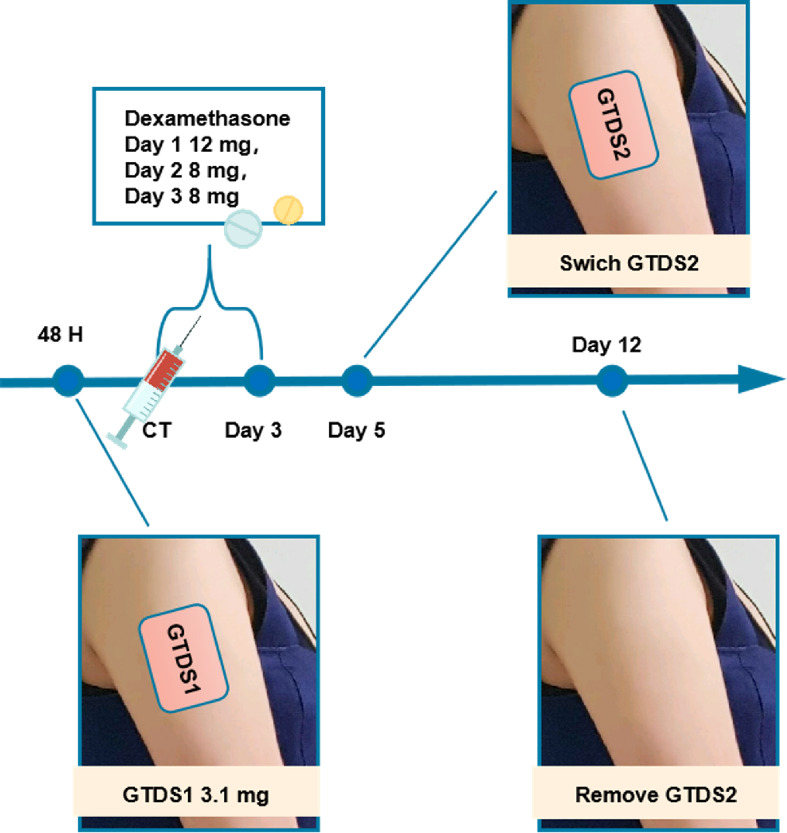
Description of Treatment Regimen GTDS1 attached to the upper arm 48 h before chemotherapy, replaced GTDS2 on day 5 and discarded GTDS2 on day 12. Dexamethasone 12 mg orally on day 1, and Dexamethasone 8 mg orally on days 2–3. Abbreviation: CT, chemotherapy; GTDS, granisetron transdermal delivery system

**Fig. 2:**
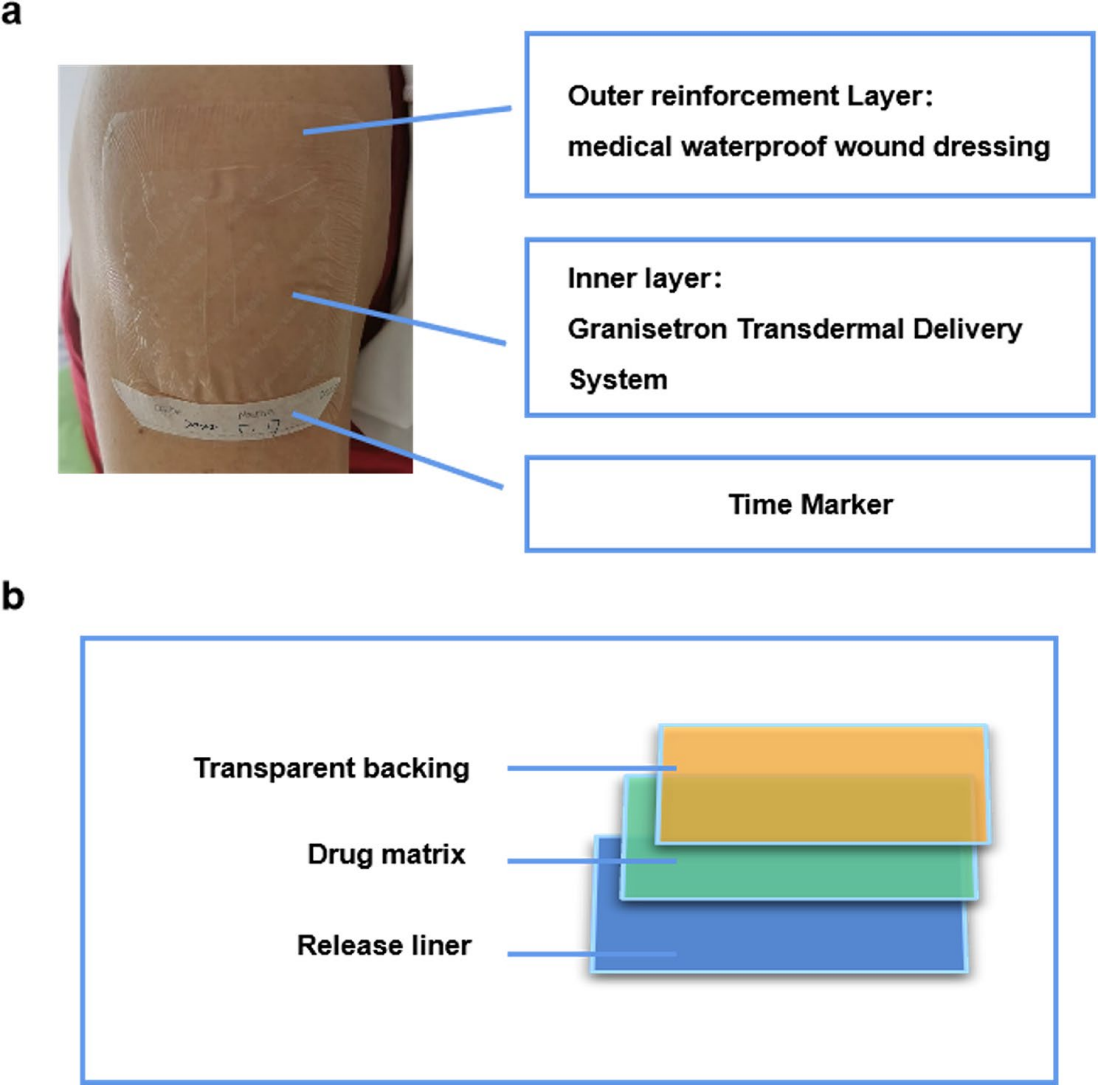
GTDS Construction Diagram The GTDS patch consists of a transparent backing, drug matrix, and release liner, with a waterproof reinforcement to prevent accidental peeling

All patients received the GTDS (3.1 mg attached to the upper arm 48 h before chemotherapy, replaced on day 5 and discarded on day 12), Dexamethasone (DEX: 12 mg oral administration on day 1, 8 mg oral administration on days 2–3). A diagrammatic description of the treatment regimen is shown in Fig. 1. The GTDS comprises a transparent backing, a drug matrix, and a release liner. The patch was reinforced with a medical waterproof wound outside to ensure it could not accidentally peel off, as shown in Fig. 2. All patients in this study were prescribed oral metoclopramide (10 mg) as an on-demand rescue medication, and all episodes of use of metoclopramide were recorded.

**Fig. 3:**
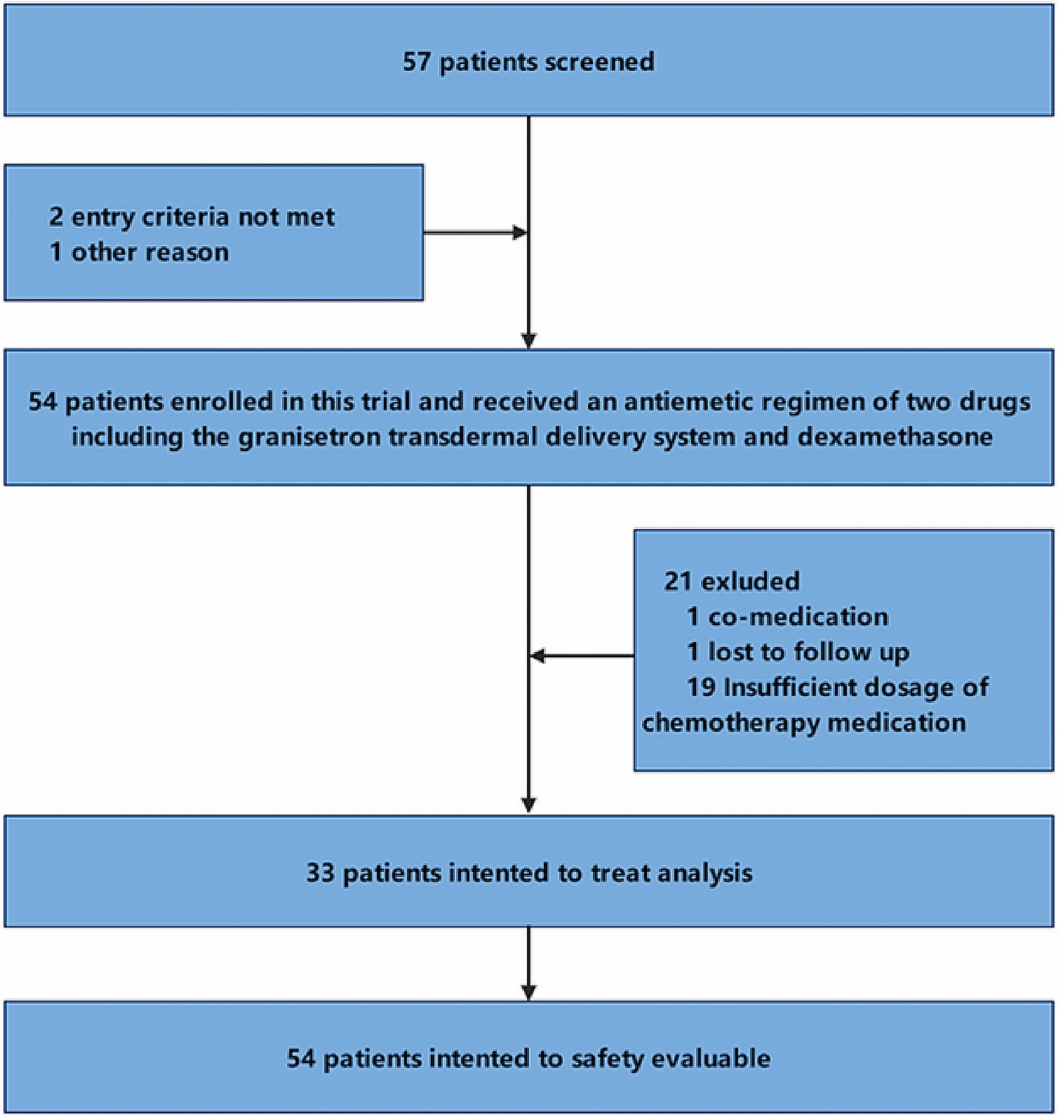
Study Flow Diagram

### Assessment

In the pre-study period, all patient demographic characteristics and medical data were recorded. All patients were followed at outpatient clinics or contacted by a research nurse by phone, recording the presence of nausea and vomiting using the Common Terminology Criteria for Adverse Events (CTCAE) version 5.0 scale (none, mild, moderate, severe).

The FLIE questionnaire consists of two domains (nausea and vomiting) [9]. In each domain, patients respond to one question about the severity of symptoms (nausea or vomiting), followed by eight questions to assess the impact of nausea and vomiting on the patient, such as eating, doing household chores, performing daily activities, engaging in leisure activities, etc. The FLIE scale utilizes a 7-point visual analogue scale (VAS) to record patient responses, with higher scores indicating better quality of life. An average item score > 6 is defined as no impact of CINV on daily life. Patients completed an 18-item FLIE questionnaire before chemotherapy and on day 6 after chemotherapy. The proportion of patients with no impact on daily life is defined as an average item score > 6 on the 7-point VAS (with a FLIE total score > 108).

### Outcomes

The primary endpoint was the complete control (CC) rate of delayed nausea and vomiting, defined as no emetic episodes, no use of rescue medication, and no nausea (Visual Analogue Scale, VAS < 25 mm) occurred during the assessment period (25 to 480 h; 20 days after chemotherapy).

The secondary endpoints were the dates of delayed complete control (DDCC), defined as the dates of no emetic episodes, no use of rescue medication, and no nausea (VAS < 25 mm) occurring during the assessment period (25 to 480 h; 20 days after chemotherapy). The complete control rate of acute nausea and vomiting was defined as no emetic episodes and no administration during the acute assessment period (0–24 h). Adverse events were graded according to the CTCAE version 5.0. The Functional Living Index - Emesis (FLIE) was applied to assess the impact of CINV on patients’ daily lives.

### Statistics

This study hypothesized that the CC rate of delayed nausea and vomiting for combined therapy using the granisetron transdermal patch and DEX would be significantly greater than that of the CC rate for standard antiemetic doublet therapy. One trial found the CC rate to be 48.7% [4]. Therefore, the null hypothesis assumed the CC rate to be 48.7%. The study was designed according to Testing One Proportion using the Exact Test to detect an improvement in the CC rate from 48.7 to 70%. Sample size calculation was conducted using PASS 15 software (NCSS LLC, Kaysville, UT, USA). We aimed to use a significance level of 0.05 and targeted a power of 76%. Using the one-sided exact test available in PASS software, the required sample size was calculated to be 29 participants. We also assumed that the dropout rate would be less than 10% over the course of the study. Therefore, we plan to enroll a total of 33 patients in this study to accommodate for potential dropouts. The patients’ characteristics, CC and CR rate, and treatment-related adverse events were summarized using descriptive statistics or reported as the frequency and proportion of the total patients. The Clopper–Pearson exact method was used to calculate the 95% confidence intervals [CI] for the CC and CR rates.

## Results

### Patient characteristics

From October 2021 through June 2023, 57 patients were screened, and 54 patients were enrolled. A total of 21 patients were excluded, 1 patient used matrine injection during chemotherapy, and 19 patients received insufficient dosage of chemotherapy medication. One patient refused to keep a diary. Therefore, the data from 57 patients, 24 women and 33 men with a mean (range) age of 63.2 (36–75) years, were analysed (Fig. 2). Details of patient enrollment, eligibility, and treatment are provided in supplement. The participants’ characteristics are shown in Table 1, including sex, age, medications or anticancer agents, and drinking habits. Most patients were male (62.3%), colon cancer was the most common type of cancer (56.6%).

**Table 1:** Patient characteristics

Characteristic	All Patients (*N* = 53)	
	*N*	(%)
Age, n (%)		
40–49 yr	9	17.0
50–59 yr	23	43.4
≥ 60 yr	21	39.6
Gender, n (%)		
Male	33	62.3
Female	20	37.7
ECOG PS, n (%)		
0	31	58.5
1	22	41.5
Malignancy, n (%)		
Gastric cancer	8	15.1
Colon cancer	30	56.6
Rectal cancer	15	28.3
Anticancer drugs		
Oxaliplatin		
117–129 mg/m^2^	12	22.6
130–145 mg/m^2^	41	77.4
Capecitabine (single oral dose)		
806–999 mg/m^2^	19	35.8
1000–1388 mg/m^2^	34	64.2
Habitual alcohol consumption, n (%)		
Yes	24	45.3
No	29	54.7
Morning sickness, n (%)		
Yes	14	26.4
No	23	43.4
No experience	9	17.0
Unknown	7	13.2

*Abbreviations* ECOG PS: Eastern Cooperative Oncology Group performance status

### Efficacy

**Fig. 4:**
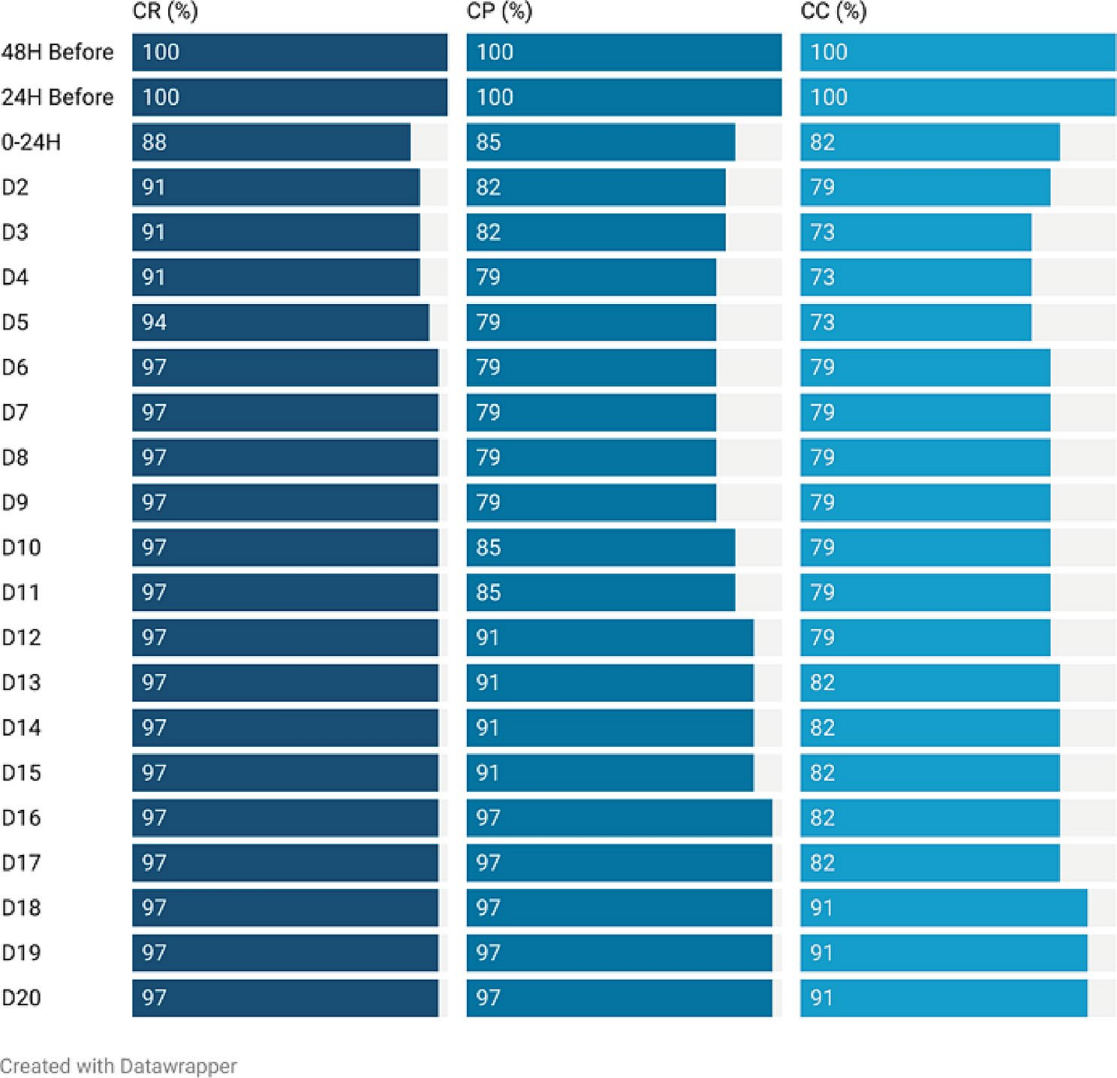
The Antiemetic Effects of Granisetron Transdermal Delivery Syst**em** This figure show the antiemetic effects from 48 h before chemotherapy to 20 day after chemotherapy. Abbreviations: CC, complete control; CP, complete protection; CR, complete response. This figure was prepared with Datawrapper (https://www.datawrapper.de/)

**Fig. 5:**
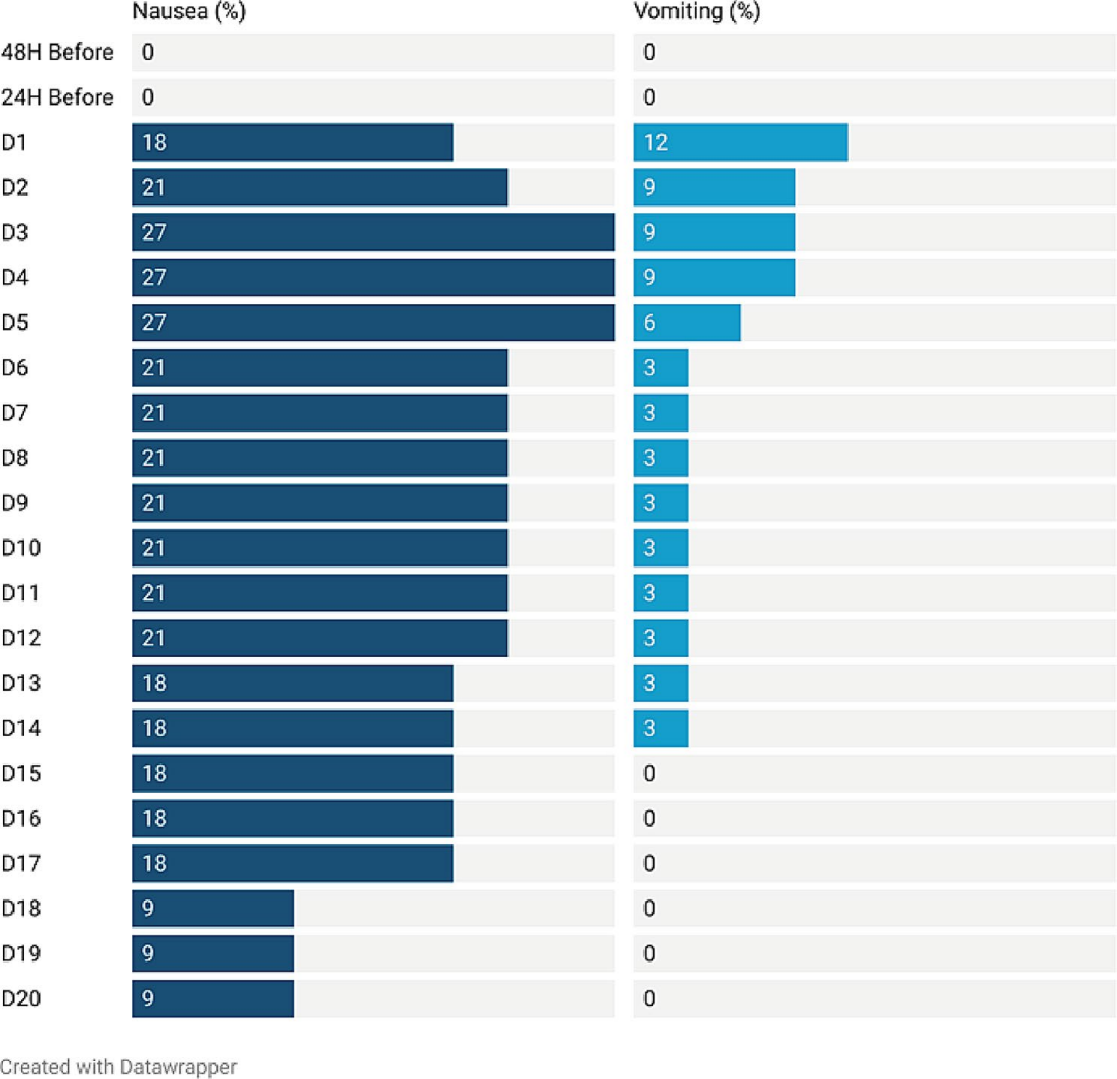
The Occurrence of Nausea and Vomiting The occurrence of nausea and vomiting from 48 h before chemotherapy to 20 day after chemotherapy. This figure was prepared with Datawrapper (https://www.datawrapper.de/)

**Fig. 6:**
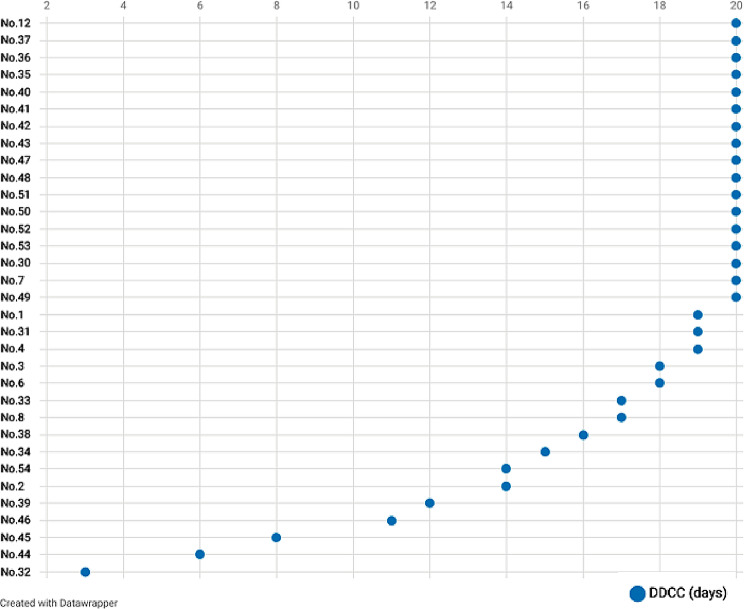
The Dates of Delayed Complete Control of Granisetron Transdermal Delivery Systems Abbreviations: DDCC, dates of delayed complete control; N, number. This figure was prepared with Datawrapper (https://www.datawrapper.de/)

For the 20-day assessment period, the complete control rate of the primary endpoints was 72.7% (95% CI 0.57 ~ 0.88). For the acute phase (0–24 h) and delayed phase (25–120 h), the complete control rates were 81.8% (95% CI 0.69–0.95) and 72.7% (95% CI 0.57–0.88), respectively. The antiemetic effects are shown in Table 2; Fig. 4. The incidence of nausea showed a trend of first rise in 3 days after chemotherapy and then decline, as showed in Fig. 5. For the secondary endpoints, the DDCC included 17.2 ± 4.5 days (Fig. 6). The DDCC of more than half patients was 20 days. Only 3 patients (10%) experienced chemotherapy interruption due to CINV.

**Table 2:** Result of the antiemetic activity during acute, delayed and continued assessment period

	Complete response (*N* = 33)			Complete protection (*N* = 33)			Complete Control (*N* = 33)		
`	N	%	95% CI	N	%	95% CI	N	%	95% CI
BC (-48 h to 0 h)	33	100	0.92 ~ 1.00	33	100	0.92 ~ 1.00	33	100	0.92 ~ 1.00
Acute (0–24 h)	29	87.9	0.77 ~ 0.99	28	84.8	0.70 ~ 0.96	27	81.8	0.69 ~ 0.95
Delayed (25–120 h)	30	90.9	0.74 ~ 0.98	26	78.8	0.60 ~ 0.89	24	72.7	0.57 ~ 0.88
Continued (121–480 h)	32	97.0	0.79 ~ 1.00	28	78.8	0.60 ~ 0.89	26	78.8	0.60 ~ 0.89

*Abbreviations* BC: Before Chemotherapy

### Adverse events

**Fig. 7:**
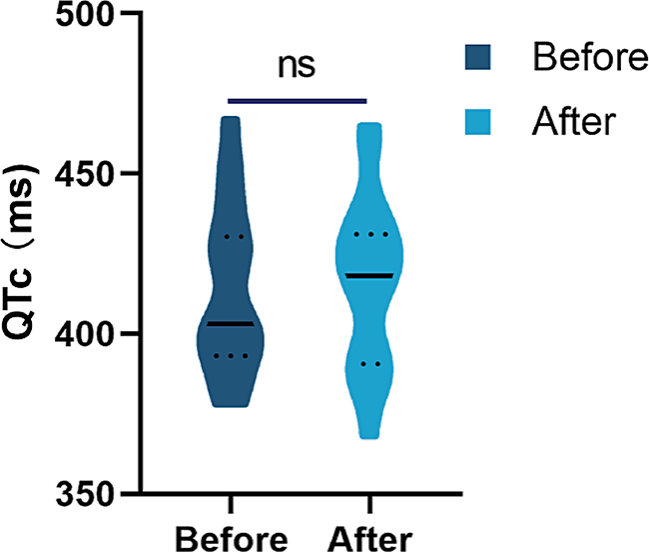
Change in QT Interval Before and After Treatment NS, not significant

The prevalence of adverse events is shown in Table 3. Using the CTCAE version 5.0, 3 (5.5%) patient reported chemotherapy-induced myelosuppression of grade 3, and 2 (3.7%) patient reported a grade 3 adverse event (diarrhea). The peak incidence of anemia was 38.9% in grades 1 and 2. Therefore, we suggest that most adverse events were related to the antitumor treatment. However, 29.6% of the constipation cases in grades 1 and 2 were related to antiemetic drugs. The difference was not statistically significant in the QT/QTc interval before and after treatment (T = 0.356, *p* = 0.724) (Fig. 7).

**Table 3:** Summary of adverse events

Adverse event (AE) by CTCAE	Grade 1	Grade 2	Grade 3	Grade 4	≥Grade 3	Any Grade
	*n* (%)	*n* (%)	*n* (%)	*n* (%)	*n* (%)	*n* (%)
Anaemia	6 (11.1)	4 (7.4)	1 (1.9)	0	1(1.9)	11 (20.4)
Neutrophil count decreased	7 (13.0)	5 (9.3)	1 (1.9)	0	1 (1.9)	13 (24.1)
Platelet count decreased	6 (11.1)	4 (7.4)	1 (1.9)	0	1 (1.9)	11 (20.4)
ALT increased	4 (7.4)	3 (5.5)	0	0	0	7 (13.0)
AST increased	5 (9.3)	3 (5.5)	0	0	0	8 (14.8)
ALP increased	3 (5.5)	0	0	0	0	3 (5.5)
BIL increased	1 (1.9)	0	0	0	0	1 (1.9)
GGT increased	4 (7.4)	0	0	0	0	4 (7.4)
Creatinine increased	1 (3.4)	0	0	0	0	1 (3.4)
Constipation	10 (18.5)	5 (9.3)	0	0	0	15 (27.7)
Diarrhea	5 (9.2)	4 (7.4)	0	0	0	9 (16.7)
Insomnia	2 (3.7)	1 (1.9)	0	0	0	3 (5.5)
Rash	2 (3.7)	0	0	0	0	2 (3.7)
Tachycardia	4 (7.4)	0	0	0	0	4 (7.4)
Peripheral sensory neuropathy	7 (13.0)	2 (3.7)	0	0	0	9 (16.7)
Hand foot Sndrome	6 (11.1)	4 (7.4)	0	0	0	10 (18.5)

*Abbreviations* ALP: Alkaline phosphastase serum; ALT: Glutamic-pyruvate transaminase; AST: Glutamic-oxaloacetic transaminase; BIL: Bilirubin; CTCAE: Common Terminology Criteria for Adverse Events; GGT: Glutamic-oxaloacetic transaminase

### **Quality of life**

**Fig. 8:**
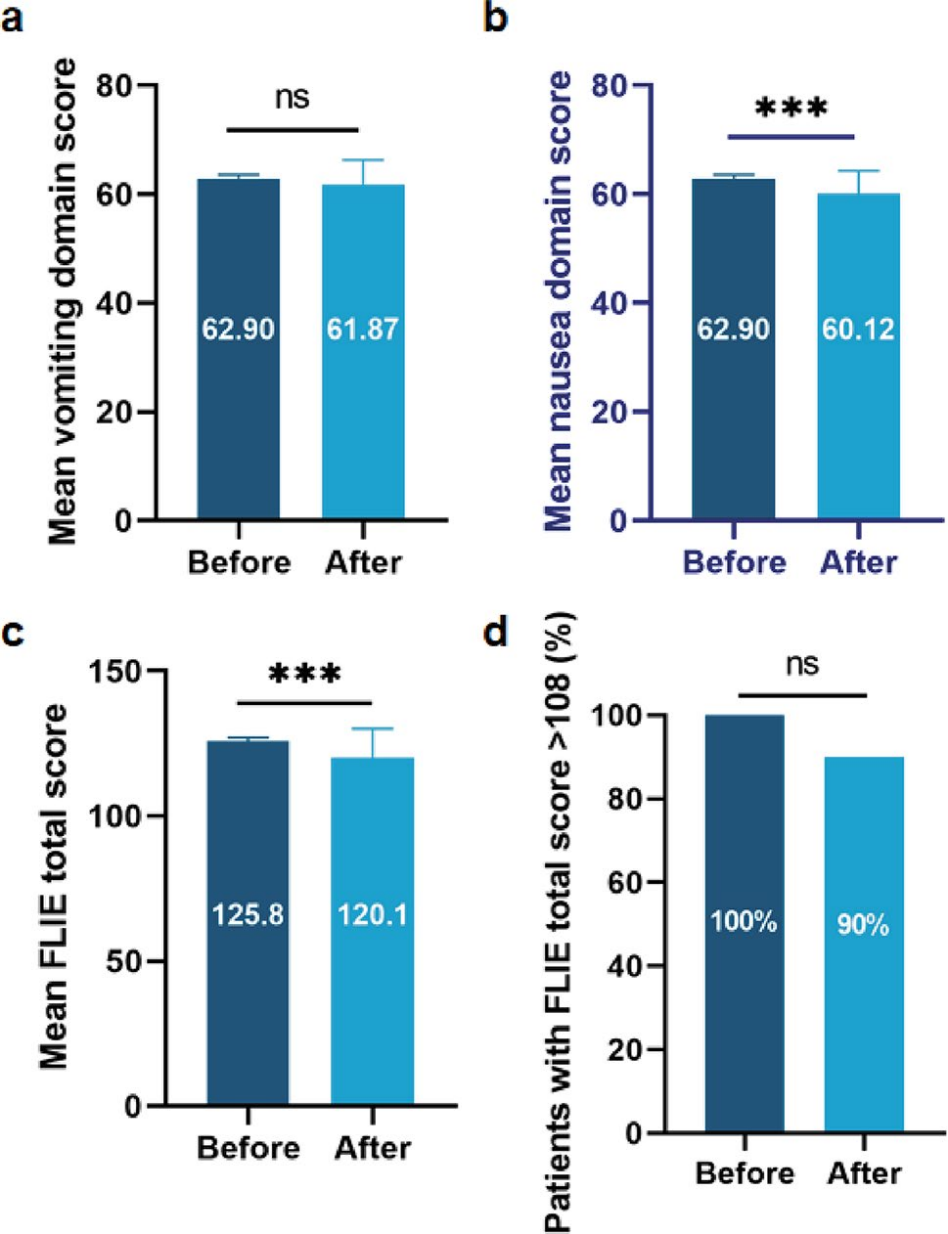
The Change of FLIE Outcome During Treatment **(a)** Contrast of mean FILE domain scores for vomiting before and after chemotherapy; **(b)** Contrast of mean FILE domain scores for nausea before and after chemotherapy. **(c)** Contrast of mean FLIE total scores before and after chemotherapy; **(d)** Percentage of patients with no impact on daily life. Error bars represent 95% confidence intervals. ***p < 0.001; NS, not significant

The results showed that the FLIE total score (mean difference = -5.727 ± 1.521; 95% confidence interval [CI] -8.750 to -2.705; *p* < 0.001; Fig. 8c), and nausea domain score (mean difference = -2.885 ± 0.5843; 95% confidence interval [CI] -4.044 to -1.726; *p* < 0.001; Fig. 8b) after chemotherapy was significantly decreased compared with those before chemotherapy. But no statistical significance was found vomiting domain score (mean difference = -0.7500 ± 0.5940; 95% confidence interval [CI] -1.928 to 0.428; *p*>0.05; Fig. 8a) before and after chemotherapy. The proportion of patients with no impact on daily life before and after chemotherapy, defined as FLIE total score > 108 was no statistical significance (*p*>0.05; Fig. 8d).

## Discussion

To our knowledge, this study is the first open-label, prospective, multi-center phase 2 trial investigating the efficacy and safety of preventive prolonged administration of the GTDS for CapeOX regimen. We found that the CC rate of delayed CINV for the 20-day assessment periods was 72.7% (95% CI 0.57 ~ 0.88). This is more than those previously reported 48.7%, and also superior to our hypothesis 70%. The CC rates during the acute phase (81.8%, 95% CI 0.69–0.95) was also better than that reported in the previous study (62.8%) [4]. Our study provides new reference for antiemetic therapy for CapeOX regimen, and it should be considered as the better antiemetic regimen for patients than traditional two-drug combination.

For another, we propose a new the observation time window which is 20-day assessment period, and define a new endpoint as DDCC. DDCC define as the dates of no emetic episodes, no use of rescue medication, and no nausea (VAS < 25 mm) occurring during the assessment period (25 to 480 h; 20 days after chemotherapy). Capecitabine plus oxaliplatin combination therapy is multi-day chemotherapy regimen. The emetogenic mechanism by CapeOX is complex. Over a 5-day period, there is also 50% incidence of nausea among patients, and full-dose chemotherapy is only 77.8% in clinical practice. ^[^ [4, 5]^]^ These data support the importance of continuous monitoring of the therapeutic efficacy of the antiemetic regimen. The participants in this study achieved the DDCC of 17.2 ± 4.5 days during the 21-day chemotherapy cycle. 51.5% of patients did not experience nausea of any grade during the delayed phase. These data show a clear antiemetic effect in CINV compared with the standard antiemetic regimen.

In this II trial we design a combined antiemetic regimen comprising the GTDS (14 consecutive days) and DEX for preventing CINV throughout the chemotherapy course. GTDS is attached to the upper arm 48 h before chemotherapy, replaced on day 5 and discarded on day 12. The design is based on the antiemetic efficacy and pharmacokinetic data of GTDS. The granisetron transdermal patch is a 6 × 8 cm.

transparent plastic patch with an adhesive-backed layer containing 34.3 mg of granisetron that releases continuously over 7 days. The results of phase III clinical trials showed a sustained steady antiemetic effect from the GTDS with a CR rate of 60–75%, equivalent to oral granisetron for 4–5 days [10]. In addition, another phase III clinical trial showed that the CC rate of the GTDS was 47.52% in moderately or highly emetogenic chemotherapy [11]. The phase I study showed the mean elimination half-life was 35.9 h. And the mean maximum plasma concentration was approximately equal to 48 h. Maximal plasma concentration of granisetron were reached 48 h, and the average concentration of GTDS over the treatment period is 2.2 ng/mL. Additionally, the mean value of concentration until patch removal is 1.1ng/mL [12]. Moreover, transdermal granisetron is relatively safe. No report has found a clinically significant effect on blood pressure, heart rate, or electrocardiogram recordings [13]. Transdermal granisetron was administered with a stable delivery without the plasma peaks and troughs characteristic of oral administration of granisetron but provided similar exposure [14]. Therefore, prolonged administration was feasible if two granisetron transdermal patches were alternated. In this study, we designed continued drug administration for 14 days based on the granisetron transdermal patch’s potential efficacy, safety, and pharmacokinetic features.

In terms of safety, phase III studies found no statistically significant difference between transdermal and oral administration of granisetron for the risk of constipation and QTc prolongation [8, 10, 11]. Our trial indicated that the granisetron transdermal patch has good tolerance and safety. No statistical difference was found in QTc prolongation before and after treatment. However, it is worth noting that the incidence of constipation was greater (27.7%) than in previous reports (6.6–8.4%) [8]. Whether prolonged administration of the granisetron transdermal patch increased the incidence of constipation remains to be determined.

The efficacy of GTDS in minimizing the negative impact of CINV on patients’ daily lives was quantitatively measured using the FILE questionnaire, which is a validated assessment tool. No statistical significance was found in vomiting domain score and the proportion of patients with no impact on daily life before and after chemotherapy. It can be seen that GTDS not only have a clear antiemetic effect, but also have the maintaining quality of life.

There are some limitations to the current study that suggest new directions for research. First, the method of prolonged administration of GTDS still needs to be improved. From Fig. 3, we can find that the CC rate was decreased from the beginning of chemotherapy and reached its lowest level on day 3. However, whether loss in antiemetic efficacy is related to the decline in drug concentration is still unknown. Second, the increased incidence of constipation remains to be confirmed. Additionally, the present study was a preliminary exploration of the clinical activity and safety of GTDS in patients with CapeOX. The current data were not mature because of the nature of the study (i.e., it was a single-arm study) and its small sample size. Further research is needed, including the design of multicenter randomized controlled clinical trials, with the current standard antiemetic treatment regimen as the control group, in order to confirm the findings presented in this study.

In summary, administering the GTDS to prevent delayed CINV is a safe alternative to antiemetics for patients of gastrointestinal malignancies treated with CapeOX.

### Electronic supplementary material

Below is the link to the electronic supplementary material.

Supplementary Material 1

## Data Availability

The datasets generated during the current study are not publicly available due to the nature of patient privacy and confidentiality constraints. Anonymized data may be available from the corresponding author upon reasonable request and with the appropriate ethical approvals from the relevant institutional review boards. Requests should be directed to Zhuchen Yan. Data will be provided in compliance with all applicable regulations and with the understanding that the use of the data will be limited to non-commercial research purposes and that all data users must adhere to strict confidentiality agreements.

## Abbreviations

GTDS: Granisetron transdermal delivery system
CINV: Chemotherapy-induced nausea and vomiting
CapeOX: Capecitabine plus Oxaliplatin
5-HT: 5-hydroxytryptamine
NK-1: neurokinin-1
FDA: Food and Drug Administration
QTc: QT interval
DEX: Dexamethasone
ECOG: Eastern Cooperative Oncology Group
CTCAE: Common Terminology Criteria for Adverse Events
FLIE: Food and Life Enjoyment
VAS: Visual Analogue Scale
CC: Complete Control
CR: Complete Response
DDCC: Dates of Delayed Complete Control
