# Supplementary material for: Prolonged administration of the granisetron transdermal delivery system reduces capecitabine plus oxaliplatin regimen induced nausea and vomiting
Source: BMC Cancer. 2024 Jul 18;24:867. doi: 10.1186/s12885-024-12616-9 (PMC11264757; doi:10.1186/s12885-024-12616-9)
Supplement: Supplementary file 1 — Supplementary Material 1 [file 12885_2024_12616_MOESM1_ESM.docx]

**THE CONSORT STATEMENT**

| **Section** |  | **Item No.** | **CONSORT 2010 statement**^1^ |  | **Item No.** | **CONSORT-Outcomes 2022 Extension**^2^ |
| --- | --- | --- | --- | --- | --- | --- |
| **Title and abstract** | | | | | | |
| Title and abstract | □ | 1a | Identification as a randomized trial in the title | □ |  |  |
|  | ☑ | 1b | Structured summary of trial design, methods, results, and conclusions (for specific guidance see CONSORT for abstracts) | □ |  |  |
| **Introduction** | | | | | | |
| Background and objectives | ☑ | 2a | Scientific background and explanation of rationale |  |  |  |
|  | ☑ | 2b | Specific objectives or hypotheses |  |  |  |
| **Methods** | | | | | | |
| Trial design | ☑ | 3a | Description of trial design (such as parallel, factorial) including allocation ratio |  |  |  |
|  | □ | 3b | Important changes to methods after trial commencement  (such as eligibility criteria), with reasons |  |  |  |
| Participants | ☑ | 4a | Eligibility criteria for participants |  |  |  |
|  | ☑ | 4b | Settings and locations where the data were collected |  |  |  |
| Interventions | ☑ | 5 | The interventions for each group with sufficient details to allow replication, including how and when they were actually administered (for specific guidance see TIDieR  checklist and guide)^3^ |  |  |  |
| Outcomes |  | 6a | Completely defined prespecified primary and secondary outcome measures, including how and  when they were assessed | ☑ | 6a.1 | Provide a rationale for the selection of the domain for the trial’s primary outcome |
|  |  |  |  | ☑ | 6a.2 | Describe the specific measurement variable (eg, systolic blood pressure), analysis metric (eg, change from baseline, final value, time to event), method of aggregation (eg, mean, proportion), and the time point for each outcome |
|  |  |  |  | □ | 6a.3 | If the analysis metric for the primary outcome represents  within-participant change, define and justify the inimal  important change in individuals |
|  |  |  |  | □ | 6a.4 | f the outcome data were continuous, but were analyzed as categorical (method of aggregation), specify the cutoff values used |
|  |  |  |  | □ | 6a.5 | If outcome assessments were performed at several time  points after randomization, state the time points used for  the analysis |
|  |  |  |  | □ | 6a.6 | If a composite outcome was used, define all individual  components of the composite outcome |
|  |  |  |  | □ | 6a.7 | Identify any outcomes that were not prespecified in a trial registry or trial protocol |
|  |  |  |  | ☑ | 6a.8 | Provide a description of the study instruments used to  assess the outcome (eg, questionnaires, laboratory tests) along with reliability, validity, and responsiveness in a population similar to the study sample |
|  |  |  |  | □ | 6a.9 | Describe who assessed the outcome (eg, nurse, parent) and any qualifications or trial-specific training necessary to administer the study instruments to assess the outcome |
|  |  |  |  | □ | 6a.10 | Describe any processes used to promote outcome data  quality during data collection (eg, duplicate measurements) and after data collection (eg, range checks of outcome data values), or state where these details can be found |
|  | □ | 6b | Any changes to trial outcomes after the trial commenced, with reasons |  |  |  |
| Sample size | ☑ | 7a | How sample size was determined | ☑ | 7a.1 | Define and justify the target difference between treatment  groups (eg, the minimal important difference) |
|  | □ | 7b | When applicable, explanation of any interim analyses and stopping guidelines |  |  |  |
| **Randomization** | | | | | | |
| Sequence  generation | □ | 8a | Method used to generate the random allocation sequence |  |  |  |
| Allocation  concealment  mechanism | □ | 9a | Type of randomization; details of any restriction (such as blocking and block size) |  |  |  |
|  | □ | 9b | Mechanism used to implement the random allocation sequence (such as sequentially numbered containers), describing any steps taken to conceal the sequence until  interventions were assigned |  |  |  |
| Implementa-  tion | □ | 10 | Who generated the random allocation sequence, who  enrolled participants, and who assigned participants to  interventions |  |  |  |
| Blinding | □ | 11a | If done, who was blinded after assignment to interventions (for example, participants, care providers, those assessing outcomes) and how |  |  |  |
|  | □ | 11b | If relevant, description of the similarity of interventions |  |  |  |
| Statistical methods | □ | 12a | Statistical methods used to compare groups for primary  and secondary outcomes | ☑ | 12a.1 | Describe any methods used to account for multiplicity in  the analysis or interpretation of the primary and secondary  outcomes (eg, coprimary outcomes, same outcome  assessed at multiple time points, or subgroup analyses  of an outcome) |
|  |  |  |  | ☑ | 12a.2 | State and justify any criteria for excluding any outcome  data from the analysis and reporting, or report that no  outcome data were excluded |
|  |  |  |  | □ | 12a.3 | Describe the methods used to assess patterns of  missingness (eg, missing not at random), and describe the  methods used to handle missing outcome items or entire assessments |
|  |  |  |  | □ | 12a.4 | Provide a definition of the outcome analysis population  relating to nonadherence of the trial protocol (eg, as a randomized analysis) |
|  | □ | 12b | Methods for additional analyses, such as subgroup analyses and adjusted analyses |  |  |  |
| **Results** | | | | | | |
| Participant flow (a diagram is  strongly  recommended) | □ | 13a | For each group, the numbers of participants who were randomly assigned, received intended treatment, and were analyzed for the primary outcome |  |  |  |
|  | □ | 13b | For each group, losses and exclusions after randomization, together with reasons |  |  |  |
| Recruitment | ☑ | 14a | Dates defining the periods of recruitment and follow-up |  |  |  |
|  | ☑ | 14b | Why the trial ended or was stopped |  |  |  |
| Baseline data | ☑ | 15 | A table showing baseline demographic and clinical  characteristics for each group |  |  |  |
| Numbers analyzed | □ | 16 | For each group, number of participants (denominator)  included in each analysis and whether the analysis was by  original assigned groups |  |  |  |
| Outcomes and  estimation | ☑ | 17a | For each primary and secondary outcome, results for each group, and the estimated effect size and its precision (such as 95% CI) | ☑ | 17a.1 | Include the results for all prespecified outcome analyses or state where the results can be found if not in this report |
|  | ☑ | 17b | For binary outcomes, presentation of both absolute and relative effect sizes is recommended |  |  |  |
| Ancillary analyses | □ | 18 | Results of any other analyses performed, including  subgroup analyses and adjusted analyses, distinguishing  prespecified from exploratory | □ | 18.1 | If there were any analyses that were not prespecified,  explain why they were performed |
| Harms | □ | 19 | All important harms or unintended effects in each group (for specific guidance see CONSORT for harms^4^) |  |  |  |
| **Discussion** | | | | | | |
| Limitations | ☑ | 20 | Trial limitations, addressing sources of potential bias,  imprecision, and, if relevant, multiplicity of analyses |  |  |  |
| Generalizabil-  ity | ☑ | 21 | Generalizability (external validity, applicability) of the  trial findings |  |  |  |
| Interpretation | ☑ | 22 | Interpretation consistent with results, balancing benefits  and harms, and considering other relevant evidence |  |  |  |
| **Other information** | | | | | | |
| Registration | ☑ | 23 | Registration number and name of trial registry |  |  |  |
| Protocol | ☑ | 24 | Where the full trial protocol can be accessed, if available |  |  |  |
| Funding | ☑ | 25 | Sources of funding and other support (such as supply of drugs), role of funders |  |  |  |
| Abbreviations: CONSORT, Consolidated Standards of Reporting Trials; TIDieR, Template for Intervention Description and Replication. | | | | | | |

**References:**

1. Moher D, Hopewell S, Schulz KF et al. CONSORT 2010 explanation and elaboration: updated guidelines for reporting parallel group randomised trials. *BMJ*. 2010;340:c869

2. Butcher NJ, Monsour A, Mew EJ et al. Guidelines for Reporting Outcomes in Trial Reports: The CONSORT-Outcomes 2022 Extension. *JAMA*. 2022;328(22):2252-2264

3. Hoffmann TC, Glasziou PP, Boutron I et al. Better reporting of interventions: template for intervention description and replication (TIDieR) checklist and guide. *BMJ*. 2014;348:g1687

4. Ioannidis JP, Evans SJ, Gotzsche PC et al. Better reporting of harms in randomized trials: an extension of the CONSORT statement. *Ann Intern Med*. 2004;141(10):781-8
